# Rebranding exercise: closing the gap between values and behavior

**DOI:** 10.1186/1479-5868-8-94

**Published:** 2011-08-31

**Authors:** Michelle L Segar, Jacquelynne S Eccles, Caroline R Richardson

**Affiliations:** 1 Institute for Research on Women and Gender, University of Michigan, Ann Arbor, Michigan, USA; 2 Institute for Social Research, University of Michigan, Ann Arbor, Michigan, USA; 3 Department of Family Medicine, University of Michigan, Ann Arbor, Michigan, USA; 4 VA Center for Clinical Management Research, VA HSR&D Center of Excellence, Ann Arbor, Michigan, USA

**Keywords:** Physical activity, behavioral branding, higher order, superordinate, goals, values, women

## Abstract

**Background:**

Behavior can only be understood by identifying the goals to which it is attached. Superordinate-level goals are linked to individuals' values, and may offer insights into how to connect exercise with their core values and increase participation in sustainable ways.

**Methods:**

A random sample of healthy midlife women (aged 40-60y) was selected to participate in a year-long mixed-method study (n = 226). Superordinate goals were measured inductively and analyzed using grounded theory analysis. Attainment Value and Exercise Participation were quantitatively measured. An ANOVA and pairwise comparisons were conducted to investigate the differences between superordinate exercise goals in attainment value. This study fit a Linear Mixed Model to the data to investigate the fixed effects of superordinate goals on exercise participation, controlling for BMI and social support.

**Results:**

Participants mainly exercised to achieve Healthy-Aging, Quality-of-Life, Current-Health, and Appearance/Weight superordinate goals. Despite equally valuing Healthy-Aging, Quality-of-Life, and Current-Health goals, participants with Quality-of-Life goals reported participating in more exercise than those with Current-Health (p < 0.01), and Healthy-Aging (p = 0.06) goals.

**Conclusions:**

Superordinate exercise goals related to health and healthy aging are associated with less exercise than those related to enhancing daily quality of life, despite being equally valued. While important, pursuing distant benefits from exercise such as health promotion, disease prevention, and longevity might not be as compelling to busy individuals compared to their other daily priorities and responsibilities. By shifting our paradigm from medicine to marketing, we can glean insights into how we can better market and "sell" exercise. Because immediate payoffs motivate behavior better than distant goals, a more effective "hook" for promoting sustainable participation might be to rebrand exercise as a primary way individuals can enhance the quality of their *daily *lives. These findings have important implications for how we as a culture, especially those in fitness-related businesses, health promotion, health care, and public health, prescribe and market exercise on individual and population levels.

## Background

Regular exercise reduces the risk of developing many chronic illnesses including cardiovascular disease, diabetes, depression, osteoporosis, etc. [1]. Women are less physically active than men, and women over 50 constitute one of the most sedentary populations in the United States [1,2]. In addition, as women age their physical activity participation decreases [3]. Physical activity, however, could benefit women in midlife in many ways. Midlife women who are physically active during menopause gain less weight and experience less stress and negative affect [4]. Unfortunately, sustaining physically active lives is not easy. While a number of interventions can help individuals successfully initiate an exercise program, most interventions have failed to show that the new lifestyle is maintained [5,6]. To date, the most commonly used public health theories have not been adequate for producing sustainable changes [7]. Moreover, most theories used in exercise research do not address the influential role that goals play in participation despite goals being central to motivation and self-regulation processes [8,9].

### Goals are Primary for Understanding Exercise Participation

The centrality of goals in behavioral pursuit has been identified within numerous theories of human behavior, across disciplines [10-13]. Goal theories posit that an individual is motivated to change their behavior because they want to reduce a perceived discrepancy between their actual state and their desired state [10,14]. Carver and Scheier (1998, 1999), leading self-regulation theorists, said that goals create the frame through which a behavior is perceived and that behavior can be understood only by identifying the goals to which behavior is attached. Moreover, statistical modeling of behavior shows that the motivation individuals feel toward a behavior is partially channeled through ***the desire one feels toward their reason or goal ***for doing that behavior [12]. These evidence-based insights suggest that it is essential to study the goals that individuals strive to achieve through exercising if we are to understand how to promote sustainable exercise behavior.

Health behavior and self-regulation are inextricably influenced by culture [15,16]. The goals individuals endorse reflect cultural values and influence motivational potential - or lack thereof [17,18]. To study these two issues, we integrated two theoretical perspectives related to goal striving, decision making, and motivation as the framework for this study. The Eccles et al., Value Expectancy Model (EEVM) is a comprehensive model, and has yielded over 30 years of research suggesting that our daily decisions and goals arise out of and are strongly influenced by our socialization within the general cultural milieu, especially related to our gender roles and perceived priorities [17]. According to the EEVM, the goals individuals select for exercising are influenced by and embed culturally-endorsed values and socialized pressures. Complementary to the "top down" perspective offered by the EEVM is a "bottom up" framework that investigates the structure of goals. This specific program of research promotes a more nuanced understanding of goals because it deconstructs goals into three distinct hierarchically-structured levels [19,20].

### Goals Have Multiple Levels

**Figure 1:**
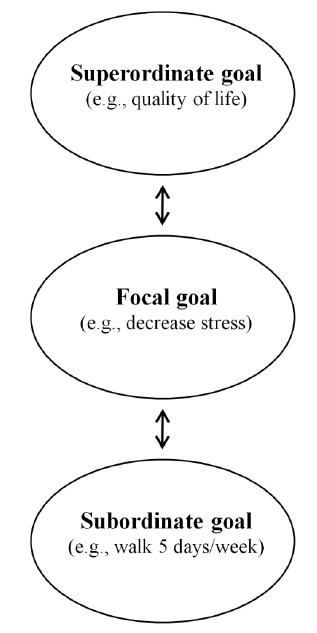
**Hierarchical Structure of Goals**.

Goals differ in level of abstraction, and are connected in a hierarchical manner [14,21]. According to Carver and Scheier's (1990) theory of self-regulation there is a three-level hierarchy of goals (Figure 1) [19]. In this model, the focal goal represents the concrete goal intention, or *what *the individual is striving to achieve with their behavior - in this case exercise (e.g., decreased cholesterol, weight loss). Below the focal level is the subordinate-level goal. This is the lowest tier in the goal structure. It represents the specific action for *how *individuals will achieve their focal goal (e.g., walking 30 minutes 5 days/week). Above the focal level is the superordinate-level goal. This goal is more abstract and represents the reason(s) *why *individuals strive for their focal goal (e.g., longevity, popularity). Investigating the different levels of exercise goals within the goal hierarchy might help us better understand how individuals have been socialized to pursue exercising.

The target of this study is the superordinate-level exercise goal. Superordinate goals, which have also been referred to as "be goals" [14], connect to the greater life values and principles that individuals hold [22]. Because they reflect individuals' idealized selves, superordinate-level goals are considered to be important self-regulatory guides for behavior, which has been modeled and tested empirically [19,21,23]. We propose that by understanding more about how exercise is connected to the self via superordinate goals we will be able to develop improved communications and methods to make exercise participation more deeply compelling to the individual; something that might improve sustainability [24,25]. Research using this hierarchical framework has had predictive validity in many different areas of research. One study, for example, reported that superordinate goals influenced hypertensive patients' beliefs, feelings and self-regulation decisions [19]; in another study they predicted volunteering for the Italian Army [20]. Research on branding has shown that consumers regulated their behavior and considered purchasing different brands of cars based on what they reported at different goal levels within their goal hierarchies [26].

### Socialization Influences Values and Goals

How individuals have been socialized to exercise is important because socialization is the process by which individuals learn what to value and pursue, thus influencing their daily priorities and decision making [27,28]. The media is an important source of socialization [29]. In reviewing the messaging about exercise by leading health organizations as well as the media, in general, it became clear that exercise is mainly promoted in society as being important for living a healthy life, preventing disease, controlling weight, and getting fit [30-33]. When exercise is written about in the popular media, body sculpting and weight loss are usually the benefits emphasized (e.g., see "Rachael Ray Shares Her Exercise Secrets - BodyWatch") [34]. In aerobics classes, the predominant messages relate to the physical body with only limited emphasis on promoting well-being [35]. This is also evident in how leading organizations promote exercise. In their women-specific "Go Red" campaign, the American Heart Association (AHA) targets "*overall health*" as the primary reason women should adopt a behavior like exercise [36].

The manner in which professionals in the health care system characterize a behavior is also likely to influence how individuals perceive and construe that behavior [37]. Exercise is also typically prescribed to patients within the health care system for its medical and health value [38]. When physicians recommend exercise to their patients it is usually discussed within the specific context of the need to diet and lose weight [39]. Moreover, in recent years, there's been a movement and campaign by leading exercise and medical organizations to explicitly brand exercise as "a medicine" [40]. Because individuals learn about behavior within a cultural context [16,17] it is crucial to understand how this socialization impacts which goals individuals strive to achieve through exercising.

We propose that individuals have been socialized to *value exercise for a limited number of health- and weight-related benefits*, and that this has influenced the particular goals they hope to achieve from exercising [18,41]. In support of this contention, previous research on the focal-goal level showed that 75% of participants reported exercise goals specifically related to health or weight [18]. In another study, 40% of the midlife female participants exercised to improve appearance and body-shape [41]. Older studies show similar results. For example, in a study of age-related reasons for exercising, younger participants (18-30 years old) endorsed physical appearance as their most important reason for exercising, while older adults (31 to 50 years old) rated both health and aesthetic benefits as primary, and more important than emotional or social benefits [42]. Thus, we hypothesize that most individuals have been socialized to consider exercise primarily for health-related and body-shaping benefits and that the majority of the current study participants will report having superordinate exercise goals related in some way to weight or health.

### Attainment Value

The EEVM is an explanatory theory for decision making and behavioral choices. How much an individual values her behavioral goal is a key predictor of behavioral decision making in the EEVM [17]. According to the EEVM, a woman is more likely to value her superordinate exercise goal if it feels personally meaningful and important to her. This construct is referred to as the 'Attainment Value' of behavior in the EEVM [27]. The higher attainment value a behavioral goal has for a woman, the more likely she is to prioritize it in her busy day [43]. A goal's attainment value is strongly influenced by cultural norms and socialized priorities [27]. Thus, because individuals have been socialized to have health as a normative core value [44], and to consider exercise as an important health behavior [31,45], it is logical that health is frequently cited as a reason for exercising [46].

Yet, despite health being a commonly endorsed value, our growing program of research suggests that health (and weight) focal-level exercise goals are not the most optimal goals for producing on-going motivation, self-regulation, and exercise behavior in women. We previously reported that focal-level goals related to health and weight were experienced as more controlling, less intrinsically motivating, and associated with less planning and participation than focal-level goals related to enhancing sense of well-being and stress reduction [9,18].

Moreover, another study conducted focus groups with women who had participated in a 12-week physical activity intervention developed for sedentary individuals within the past three years. The authors sought to identify in what ways those who stayed active differed from those who dropped out. They reported that the participants *who did not adhere *were motivated to exercise in order to lose weight [47]. In contrast, those *who did adhere *exercised specifically to enhance their daily life. These findings suggest that the goals and objectives individuals have for exercising influence whether they maintain it. We challenge the presumption that promoting exercise primarily for health benefits and weight control is ideal for producing sustained exercise behavior, and hypothesize that participants reporting superordinate exercise goals related to health or weight will report lower attainment value for those goals and will also participate in less exercise than participants who report exercising with superordinate goals related to enhancing the quality of their daily lives.

### Research Objectives

We have three study aims: 1) to identify and investigate the content of midlife women's superordinate exercise goals; 2) to identify which superordinate exercise goals are most highly valued; and 3) to identify which goals predict the most exercise participation over time.

## Methods

### Sample

A random sample of women (aged 40-60y) was selected out of the total population of female employees at one Midwestern university using records from the Human Resource Department. Inclusion criteria were: being between 40 and 60 years old, working in clerical jobs, and having Internet access and an e-mail account. This research aimed to understand optimal superordinate exercise goals among midlife women who work full time.

### Study Design and Procedure

We used a mixed-method longitudinal study design, and collected data at three time points over one year. Baseline data were collected by mail, and the two follow-up surveys (one-month post and one-year post) were conducted on-line. The independent variables, superordinate-level exercise goal and attainment value, were collected at baseline and the exercise participation data were collected at all three data collections. To control for seasonal variation, baseline and one-month data collections occurred during the fall (September/October and October/November) as did the follow-up one year later (September-November). Human Resources provided the first author with contact information of those randomly selected who matched study criteria from a database query, and potential participants were mailed a study packet. Participant compensation was based on principles of persuasion and tiered to increase compliance [48]. Participants received a $5-$20 gift certificate based on fulfillment of study participation criteria. (For more details on our study recruitment strategy please contact the first author.) Study participants not returning their baseline surveys received e-mail inquiries on days 7, 14, and 21; thereafter, they were considered non-responders. All data were collected between September 2004 and November 2005. The University of Michigan Institutional Review Board approved this study.

### Measures

#### Superordinate Exercise Goals

The Superordinate Exercise Goal was measured at baseline. This measure was based on a method previously validated [19]. This inductive, qualitative measurement technique, referred to as "laddering," was originally developed in a commercial setting to discern individuals' motives for purchasing [49]. It is an elicitation procedure whereby participants are first asked to identify their most concrete goal ("focal-level") for exercising (to lower cholesterol, lose weight, etc.), and later to move to a more abstract level for explaining *why they care about achieving that concrete goal*. Thus, in order to measure individuals' Superordinate Exercise Goal, first we determined their focal exercise goal. For more information on participant's focal-level exercise goals see Segar, et al., (2007).

After participants selected their focal-level goal for exercising, they were informed: "Some of our goals exist in isolation, but most of our goals are usually undertaken as a part of a larger, longer-term superordinate goal. For example, Becky's most important goal for exercising is disease prevention. However, this goal is really in service of her superordinate goal to live a long and healthy life." Following this information, participants were requested to "Please write in Box A the most important exercise goal that you previously gave us. Then ask yourself: Why is this exercise goal important to me? What do I hope it will give me? Write the answer in Box B." This measure allowed us to obtain idiosyncratic responses that were later coded and placed into thematic categories.

#### Attainment Value

Attainment Value was measured at baseline by averaging four items (importance, value, being worthwhile, and meaningful) that assessed the value of participants' superordinate exercise goal within the context of their *other *life goals. For example, participants were asked: Compared to the other goals you have for yourself in life, how *worthwhile *is your superordinate exercise goal? [27,50]. Responses ranged from 1 (Much less important) to 7 (Much more important). The Attainment Value scale had adequate internal consistency (α = 0.91), and the mean of this scale was 5.8 (SD = 1.1). Higher scores indicate higher levels of attainment value.

#### Exercise Participation

Exercise Participation was assessed using a modified version of the Godin Leisure-Time Exercise Questionnaire (GLTQ) [51]. The GLTQ has been used successfully across diverse populations and has a reported test-retest reliability in adults of 0.74 [52]. The GLTQ is a one-week recall instrument that assesses light, moderate, and vigorous exercise separately. Combining all three of these intensity levels creates a summary score. To assess light, moderate, and vigorous exercise, individuals were asked to estimate how many times they participate in each activity listed during a typical seven-day period. Participants wrote down the typical number of sessions per week and minutes per session that they participated in each activity listed. The total exercise summary score was obtained by multiplying each level by the METs that reflected its intensity (mild/light = 3; moderate = 5; strenuous/vigorous = 9), after which all three levels were summed. Higher scores indicated higher levels of exercise participation. The correlation between baseline GLTQ and one-year GLTQ was 0.74 in this sample. The mean GLTQ across all three data collections was 28.8 (SD = 14.1).

#### Body mass index

BMI was calculated as the ratio of study participants' self-reported weight (kg) to self-reported height squared (m^2^) [53].

#### Social Support

Social Support was measured using a Likert-type scale. Participants answered the following two questions from (1) Not at all to (7) A lot: "To what extent does your family support you exercising?" and "To what extent do your friends support you exercising?" An index of Social Support was created from the mean of these two items. Inter-item reliability was adequate, α = 0.82. The average score was 4.7 (SD = 1.8). Higher scores indicate higher levels of social support.

### Analyses

#### Qualitative analyses

The first objective of this research was to identify the content of our study participants' Superordinate Exercise Goal using grounded theory analysis. Qualitative methods are ideal for exploring substantive issues about which little is known [54]. The coding process was iterative, and initiated with putting the goals into as many micro-level categories that could be identified. Then these micro-level categories were aggregated into macro-level goal categories based on similarity across broad topics. The first author used constant comparison techniques to place the participants' Superordinate Exercise Goals into meaningful categories. As a new theme emerged, a new category was created until all of the participants' goals were coded. Goals that appeared similar in content but that were consistently worded in different ways were placed into different categories. This conservative coding strategy aimed to prevent combining groups that might be inherently different in some way, as suggested by their differing language choices. (See Results for an example.) A second coder was trained in the coding rules and free-sorted responses. Although there was high agreement (82%), we were not satisfied. Discrepancies were discussed to refine the categories and coding rules. Another coder was trained in the coding rules and free-sorted responses. Inter-rater reliability was assessed using the Kappa coefficient. There was high agreement between coders (95%), with a Kappa coefficient = 0.94. All disagreements about category placement were resolved through discussion. (For more details about the qualitative analysis please contact the first author.)

#### Quantitative analyses

We fit a Linear Mixed Model (LMM) to the exercise participation data collected at three time points over one year (baseline, post, and follow-up). The LMM investigated the fixed effects of time, superordinate goals, BMI, and social support on participation, using the exercise random subject effects to account for within-subject correlation of the repeated measures [55]. There were 226 participants included in the LMM because they had data collected from at least one time point. After fitting the LMM, statistical assumptions were checked, and violations of these assumptions were addressed by transforming the dependent variable (Exercise Participation) into the square root of the original measure.

We used a Satterthwaite approximation for the denominator degrees of freedom because we were fitting a model to correlated (longitudinal) data, and the F-test statistics in this case do not follow an exact F distribution [56]. Multiple pairwise comparisons using the least significant difference (LSD) procedure were conducted to identify significant differences between participants' superordinate exercise goals. Standardized effect sizes (delta, Δ) for the paired comparisons were calculated according to recommendations [57,58]. We controlled for body mass index (BMI) and social support in this analysis because the literature suggests that they can influence women's participation [59,60]. Because the results of the LMM permit making inferences related to between-subject variance, it is an ideal analysis to use when doing person-centered research such as this.

An ANOVA and pairwise comparisons were conducted to investigate the differences between superordinate exercise goals in attainment value. Standardized effect sizes (partial eta-squared, η_p_^2^) for the paired comparisons were calculated in SPSS (version 13.0).

## Results

### Sample

Out of the sample population of 843 employees, 400 participants were randomly selected. Fifteen out of the 400 individuals were ineligible to participate (took the pilot survey, were administrators involved in the study, or were no longer employed by the University), leaving a sample size of 385. The response rate for the baseline survey was 71% (n = 275). See Table 1 for baseline demographics. There were no differences between the study responders and non-responders in income, education, ethnicity, and age. The majority of the baseline respondents completed the post survey (97%, n = 268), and 87% (n = 239) completed the follow-up survey.

**Table 1:** Baseline Demographics (N = 275)

Age (Mean)	49.9 (5.4)
BMI (Mean)	28.0 (6.4)
Education (%)	
High School or GED	10.5
Some College	38.0
Technical College	5.5
College Degree	36.4
Grad/Prof Degree	9.1
Missing	0.4
Marital Status (%)	
Married	62.5
Living with partner	4.4
Separated	1.1
Divorced	20.0
Widowed	2.2
Single	9.8
Household Income (%)	
< $20,000	0.7
$20,000-$60,000	38.5
$60,001-$100,000	38.9
$100,001-$124,999	10.5
$125,000+	6.9
Missing	4.4
Ethnicity (%)	
African American	5.1
Asian	2.2
European American	89.5
Latina	1.1
Mixed Ethnicities	1.5
Missing	0.7

#### What Superordinate Exercise Goals do Midlife Women Have?

Nearly all participants (n = 259) filled out superordinate exercise goals. Seven distinct Superordinate Exercise Goal categories emerged from our inductive, qualitative analysis. The first category was Healthy Aging (n = 93, 36.0%). We placed goals in this category that listed things like "pain free old age" and "live long and healthy." The second category Current Health (n = 53, 20.0%) had goals like "lower cholesterol" and "healthy lifestyle." The difference between the Current Health category and the Healthy Aging category is that the emphasis in Healthy Aging was on health and functioning in *the future *not the present. While both categories emphasized health, we wanted to investigate whether "current" or "future" health goals had distinct effects.

The third category, Weight/Appearance (n = 22, 8.5%), had goals such as "lose weight" and "feel better about my appearance." The fourth category was How I Look and Feel (n = 13, 5%). We separated those in the How I Look and Feel category from those in Weight/Appearance because their wording was very different. Those in the former group consistently and identically wrote their goal using the specific terms "how I look and feel," which indicated they cared about both benefits, and this was distinct from those in Weight/Appearance. The fifth category, Quality of Life (n = 57, 22.0%), had goals such as "sleep better" and "feel centered." The sixth category, About Myself (n = 9, 3.5%), contained goals indicating they were targeting positive feelings about themselves rather than experiences, per se (e.g., "to feel good about *myself*"). Participants were placed in this group if they specifically wrote down goals that referred to impacting some aspect of "myself." The seventh category, Mixed (n = 12, 5.0%) had goals that did not fit into any of the other categories (e.g., "serving God"). As predicted, the majority of participants had goals related to health or weight.

The participants in the How I Look and Feel, About Myself and Mixed groups were not included from the subsequent quantitative analyses because of their small sample sizes. We only made predictions for the quantitative analyses with the goal categories we had prior experience researching (i.e., goals related to "quality of life," "appearance/weight," and "current health" [9,18,41]. Because we had no prior experience with goals related to "healthy aging," we had no specific hypotheses to test, and so we made no predictions related to participants with "Healthy Aging" goals.

#### Which Superordinate Goals are Associated with the Highest Attainment Value?

**Figure 2:**
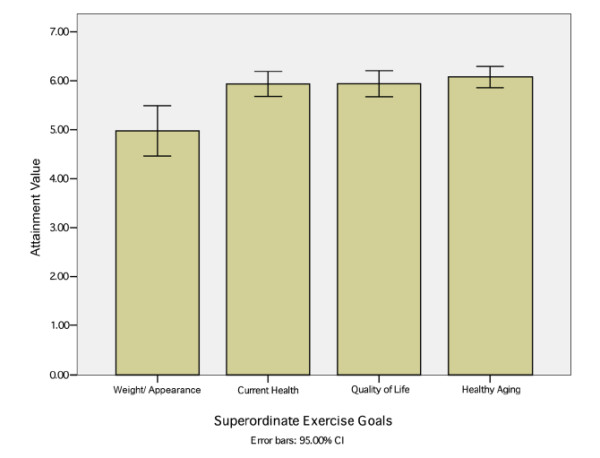
**Mean Attainment Value by Superordinate Exercise Goal**.

There was a significant difference in Attainment Value by type of goal, *F *(3, 221) = 6.7, p < 0.001, η_p_^2 ^= 0.09. As predicted, the participants with Quality of Life exercise goals valued their superordinate exercise goal significantly *more *than those with Weight/Appearance goals (*p *< 0.001, η_p_^2 ^= 0.06). Contrary to our predictions, Attainment Value was exactly the same between participants with Quality of Life goals and those with Current Health goals. Although not predicted, participants with Healthy Aging superordinate goals valued their goals equally high as those with Current Health and Quality of Life but significantly *more *than participants with Weight/Appearance superordinate goals (*p *< 0.001, η_p_^2 ^= 0.08). See the mean Attainment Value scores in Figure 2.

#### Which Superordinate Goal Predicts the Most Exercise Participation Over Time?

A linear mixed model analysis indicated significant differences between the Superordinate Exercise Goals, *F *(3, 214.5) = 3.1, *p *= 0.02 on Exercise Participation over time (i.e., baseline, one-month, and one-year post-baseline), controlling for the effects of BMI and Social Support. There was no significant main effect for either time on participation or for the time-by-goal cluster interaction. BMI *F *(1, 214.5) = 12.7, *p *< 0.001 and Social Support *F *(1, 214.2) = 18.8, *p *< 0.001 significantly predicted exercise participation over time. Participation was highest among individuals with Quality of Life superordinate goals, and lowest among those with Weight/Appearance goals. Having a lower BMI and higher social support was associated with greater exercise participation.

**Figure 3:**
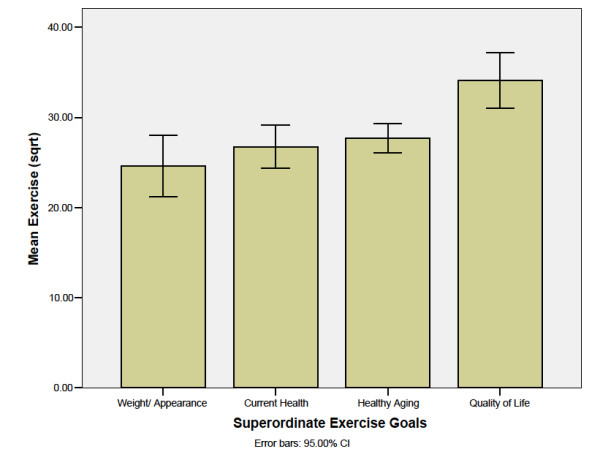
**Mean Exercise Participation Over Time by Superordinate Goal**.

As predicted, the participants with Quality of Life superordinate exercise goals exercised significantly *more *(34% more) than those with Weight/Appearance goals (*p *< 0.01, Δ = 0.55). As predicted, participants with Quality of Life goals exercised significantly *more *(25% more) than those with Current Health goals, (*p *< 0.01, Δ = 0.44). As predicted, there was no difference in Exercise Participation between participants with Weight/Appearance and Current Health goals. Although not predicted, participants with Current Health superordinate goals exercised the same amount as those with Healthy Aging goals and there was a trend showing that participants with Quality of Life goals exercised 15% *more *than those with Healthy Aging goals (*p *= 0.06, Δ = 0.29). See Figure 3 for the adjusted means of Exercise Participation with standard error bars.

## Discussion

Virtually all of the research on exercise goals has investigated the "focal-goal" level [9,61]. Yet, superordinate-level goals are thought to contribute to a more profound and lasting motivational experience than focal-level goals [14]. Because superordinate goals reflect the principles that individuals value [14], researching these higher-level goals may illuminate how exercise fits into individual's greater life objectives and their personal goal structures [10]. This is the first study to qualitatively assess the content of midlife women's superordinate exercise goals and investigate quantitatively *which *superordinate goals are most valued and most predictive of greater exercise participation over time. The majority of participants reported superordinate exercise goals related to their health in some way, but less than 25% of participants mentioned goals related to enhancing quality of life.

That such a small proportion reported quality-of-life superordinate exercise goals is concerning given that participants with Quality of Life goals exercised between 15% and 34% *more *than those with other types of goals. In general, as individuals age, they are more interested in obtaining subjective well-being experiences from physical activity [62]. This lower prevalence of quality-of-life goals may simply reflect that women have *not been socialized to consider *exercise as an effective way to enhance the quality of their daily lives. In contrast, that the majority of participants listed health or healthy aging superordinate exercise goals probably represents their socialization to exercising [17], given that these goals reflect the typical way exercise has been promoted within culture.

It is easy to recognize that the dominant messaging about exercise and physical activity, for both women and men, has promoted physical activity primarily for the health and/or weight control benefits [33,35,36]. Furthermore, exercise is typically prescribed to patients for its medical and health value rather than as a good way to enhance mood or quality of life [38]. When physicians recommend exercise to their patients it is usually discussed within the specific context of the need to diet and lose weight [39]. This makes losing weight the purpose for exercise.

In recent years, leading organizations like the American Heart Association (AHA) have developed health communications that promote quality of life alongside the health and longevity benefits of exercise: "*You'll feel better and your life depends on it*." [45]. Yet, the dominant messaging in their communications still emphasize disease prevention and life expectancy. In addition, the AHA's women-specific promotions have maintained their primary focus on heart health as the reason for participating in health behaviors like exercise: "Go Red BetterU is a FREE 12-week online nutrition and fitness program that can *makeover your heart*" [36]. In addition, a recent 2010 American Cancer Society (ACS) campaign, "Choose You," encourages women to put their *own health first *in the fight against cancer [63,64], also clearly touting disease prevention as the main reason women should adopt a health behavior like exercise.

The role of leading organizations like the ACS and the AHA is to improve the health of individuals. **Yet, we suggest that a health-related organization's primary goals may be very different than, and possibly incompatible with, the specific messaging that is most engaging and persuasive to the end user**. While other research has called for shifting the focus and promotion of exercise from body weight to health [65], these and other data suggest that promoting "health" as the main motivation to engage in exercise may also not be the most strategic message to facilitate optimal engagement and participation among individuals [9].

**The dominant messaging about exercise seems to have created a "behavioral branding" problem**. Branding is a process that purposefully aims to influence how individuals perceive, think about, and expect from a particular product, service, organization, and even a country or a person [66]. In other words, branding refers to creating an imprint of specific associations and expectations in someone's mind regarding an object or concept. Branding is a marketing concept and not one frequently discussed in the behavioral medicine, public health, and exercise literatures. Yet, the end result of branding is simply a socialization process that creates particular schemas for and expectations about something.

We suggest that the specific socialization to exercise that individuals have had through the media, health care, and society in general has explicitly branded exercise primarily as a vehicle that promotes "weight loss," "health benefits," and "disease prevention." These desired outcomes from exercise are clearly not negative! Yet, promoting exercise primarily within health care and society as a method to "improve health" or to "be thinner" might inherently foster a feeling of compliance instead of autonomy toward exercising because cultural expectations and pressures undergird these specific goals [27,31,67].

Many consider "health" to be an *autonomous *outcome to strive for and an exercise goal specifically [61,68], as we had thought it would be before our previous research [18]. However, we now argue that exercising to achieve health benefits medicalizes exercise and reflects normative pressures for what is idealized in our culture, making exercise a moral imperative, something else that we "should" be doing [31,69]. There is an important distinction between what values a culture fosters in its members and whether these values are congruent with human psychological needs and optimally motivate individuals [70]. Thus, while the societal branding of exercise has successfully been internalized by most, it may have inadvertently created a compliance-oriented brand of exercising.

Feeling controlled toward a specific behavior (e. g, feeling that one "should" do it), instead of feeling autonomous towards it (e.g., what is personally important and/or satisfying), leads individuals to feel pressure to "comply," things that are known to undermine goal pursuit and behavioral sustainability [71,72]. If the societal branding of exercise results in individuals feeling a controlled or extrinsic regulation toward exercising (instead of autonomy) than we can consider this to be non-optimal for improving population-level physical activity participation [18].

Extrinsic motives, in general, are thought to lead to poorer psychological well-being compared to intrinsic ones [25]. In addition, avoidance goals, those that focused on avoiding a negative state, have even been associated with negative physical symptoms [73]. Socialization to exercising in our culture and especially within health care has *emphasized *the use of exercise specifically to *avoid *poor health and chronic illness [40]. Thus, while counterintuitive, exercising with health goals, especially those that aim to avoid a negative state such as illness, may not be quite as healthy as one would hope.

Moreover, the relatively recent campaign devised by the prestigious American College of Sports Medicine and supported by many leading international organizations (the American Medical Association, Exercise and Sports Science Australia, the President's Council on Physical Fitness and Sports, etc.) promotes and explicitly brands "exercise is medicine" [40], something that exacerbates this problematic branding of exercise. If clinicians analogize exercise to "taking a pill" or "medicine" when speaking to their patients it may further attenuate participation, given the well-documented low adherence rates to prescription medication [74,75].

**These data also suggest that what an individual espouses as important does not necessarily translate into behavior**. It is logical and commonly thought that placing a high value on health will motivate individuals to practice health behaviors [76]. Moreover, other research suggests that health is highly endorsed as a reason for exercising [62,77]. Yet, despite all three groups equally valuing their goals, participants with exercise goals related to Current Health and Healthy Aging participated in significantly *less *exercise over time than those who had Quality of Life goals. This discrepancy is important to explore.

One explanation for the discrepancy between what one says they value and what they do could be that when women exercise "for health," "healthy aging," or "weight loss" they do not receive quick, if any, concrete feedback that they are achieving their main goal for exercising. Research shows that individuals disengage from pursuing goals when they do not receive sufficient feedback that they are making progress [14]. Furthermore, individuals have a tendency to choose smaller, immediate rewards over larger ones that occur later in time, especially when self-control is involved [78-80]. Thus, larger delayed rewards for exercising, like staying healthy or preventing illness, may not be as motivating or provide as good of feedback as smaller, immediate rewards, like improving mood or decreasing stress [81,82].

By shifting our paradigm from medicine to marketing, we can glean insights into what we might be missing in our traditional promotion of exercise. Increasing participation among individuals in sustainable ways might be a question of improving how we market and "sell" exercise through principles such as branding [66,83]. Instead of promoting the end points that clinicians, business, and government care about achieving from having individuals exercise (e.g., "improved health" in service of health care savings), health communications might become more meaningful and persuasive if they were based on the exercise benefits *that will be most compelling to individuals *[20,84,85].

Reading the language participants used to describe their superordinate goals offers insight into why exercising to enhance quality of life may trump health-related motives. Quality of Life participants wrote, "Being centered," "being balanced and relaxed," "feeling good," and "happiness" as some superordinate exercise goals. Given women's constant juggling of roles and responsibilities, it is no surprise that they want their limited leisure time to represent "relaxation," "personal freedom," "lack of constraints," and "self-determination" [86,87].

**We propose that it would be strategic to rebrand exercise as a primary method to enhance aspects of daily quality of life ***(e.g*. through social marketing, advertising, programming, and prescribing practices). Rebranding exercise with this new, in-the-moment purpose emphasizes the *immediate benefits*, such as stress reduction and increased vitality, and may also trigger individuals to appreciate the *downstream benefits *that enrich daily living (e.g., being a patient parent, enjoying life, creativity and focus at work, etc.). Striving to attain these personally meaningful and self-determined benefits might better promote well-being, engagement, and on-going participation [25,72,88,89].

**Exercise that specifically aims to enhance aspects of daily living might optimize the value of exercising and make it *more compelling *for women to fit into their busy schedules and stressful lives **[90,91]. In support of this idea, we previously reported that midlife women who exercised with focal-level goals aiming to improve the quality of their lives through reducing stress and enhancing well-being *planned *physical activity into their lives more frequently and reported higher participation levels over one year compared to those with focal-level health or weight-loss exercise goals [9]. Another study using a different design, sample, and methods also found that exercising for more autonomous goals predicted greater exercise participation and that this relationship was fully mediated by greater self-regulation strategies like planning [8]. These study findings suggest that exercise might most effectively compete against other daily goals and responsibilities if its primary purpose aims to enhance individuals' daily living experience in noticeable, pertinent, and significant ways [9,92].

Our rebranding recommendation could be considered a form of "reward substitution," a strategy from the field of behavioral economics to improve adherence by switching the motive for a behavior away from distant rewards like disease prevention to immediately-experienced incentives like increased energy [82,93,94]. Statistical modeling shows that motivation for a behavior is partially channeled through *the desire an individual feels toward their behavioral motive *[12]. **Thus**, **we need to pay much more attention to our population-level messaging about *the reasons why *individuals should take time out of their busy days to exercise**.

One important limitation of this study is that the findings cannot be generalized outside of this specific demographic of mostly white women who worked full time in the United States. Which exercise goals will most effectively motivate participation should vary across the lifespan with changes in roles, responsibilities, and priorities [27,77,95]. Thus, as midlife women age and reach retirement, see aging loved ones develop chronic conditions, and have more time to spend in leisure activities, exercise to produce good health and healthy aging might become more compelling to fit into their lives.

In addition, "health" might *mean *different things and be differently valued in distinct cultures, especially ones that have a health care system different than the United States. Thus, exercising *for health *might be experienced as more intrinsic and autonomous in different countries, as others have reported [96,97]. Studying which types of exercise goals are associated with more controlled or autonomous motivation and participation is an exciting new area of study [18,98]. The findings from this emerging literature should help inform how, as a society, we can better promote physical activity and exercise participation to better engage individuals and make it more compelling to sustain.

It should also be noted that our method for eliciting participant's superordinate goals asked them to identify only one instead of an exhaustive list of goals. While this inductive measurement method is a study strength because individuals can have multiple goals for exercising (including both autonomous and controlled), this strategy is also a weakness because it may underestimate a more complex relationship between numerous higher order goals and participation.

Using self-reported exercise data is an important limitation of this study because it is often over-reported [99]. Given that the aim of this research was to assess relationships within the data, however, there is no reason to think that those who over-reported were not evenly distributed between the superordinate goal categories. Over-reporting should also not affect the associations between the Superordinate Exercise Goal variable and the outcomes variables.

This study focused on which types of goals and messaging may most effectively persuade individuals that physical activity creates concrete and discernable value so they will feel compelled to fit it into their busy days. Type of goal may be one of many important factors that influences women's participation. Others facilitating factors include increasing women's comfort with and skills for making their own self-care a high priority, self-regulation techniques, social support, and family-friendly facilitates [59,90,100,101].

This study has many strengths. It utilized a longitudinal design over one year. It also randomly selected participants from the sample population and had excellent baseline response rate and retention of participants across the study. It was a person-centered, idiographic approach to understanding differences between individuals with similar types of goals on producing exercise behavior over time. By implementing a person-centered strategy for investigating sustained behavior the research question can go beyond making generalizations from the mean response of *variables *to the mean response of *individuals *[102]. The benefit of such a methodological strategy is that the findings have direct translation into application and improved external validity, something that has been lacking in the field of behavioral medicine [103]. Moreover, using quantitative and qualitative methods is another strength because mixed-method designs produce a more comprehensive understanding, especially of a new topic [104].

## Conclusions

Behavior can only be understood by identifying the goals to which it is attached [14]. This research adds to the emerging literature on how superordinate goals influence behavior. Our data suggest that superordinate exercise goals related to health and healthy aging are associated with less exercise than those related to enhancing daily quality of life, despite being equally valued. Individuals have been socialized to perceive and value exercise primarily as a vehicle to promote health, prevent disease, and lose weight [18,105]. While important, these types of benefits might not make exercise compelling enough to successfully compete against other daily responsibilities and priorities [43,100]. Because immediate payoffs motivate behavior better than distant goals [81,82], a more effective "hook" for promoting higher participation levels might be to rebrand exercise as a primary way individuals can enhance the quality of their daily lives [90,106]. These findings have important implications for how we as a culture, especially those in fitness-related businesses, health promotion, health care, and public health, prescribe and market exercise on individual and population levels.

## Competing interests

MLS would like to disclose that she has a consulting and training company and coaches women in how to sustain self-care behaviors and physically active lives (http://www.michellesegar.com). JSE and CRR have no competing interests to declare.

## Authors' contributions

MLS conceived of the study. MLS, JSE, and CRR participated in the study design and coordination, performed the statistical analysis, and helped to draft the manuscript. All authors read and approved the final manuscript.
